# Visual search under physical effort is faster but more vulnerable to distractor interference

**DOI:** 10.1186/s41235-021-00283-4

**Published:** 2021-03-12

**Authors:** Hyung-Bum Park, Shinhae Ahn, Weiwei Zhang

**Affiliations:** 1 Department of Psychology, University of California, Riverside, USA; 2 Department of Psychology, Chungbuk National University, Cheongju, Korea

**Keywords:** Handgrip, Singleton search, Arousal, Inhibitory control, ex-Gaussian

## Abstract

Cognition and action are often intertwined in everyday life. It is thus pivotal to understand how cognitive processes operate with concurrent actions. The present study aims to assess how simple physical effort operationalized as isometric muscle contractions affects visual attention and inhibitory control. In a dual-task paradigm, participants performed a singleton search task and a handgrip task concurrently. In the search task, the target was a shape singleton among distractors with a homogeneous but different shape. A salient-but-irrelevant distractor with a unique color (i.e., color singleton) appeared on half of the trials (*Singleton distractor present* condition), and its presence often captures spatial attention. Critically, the visual search task was performed by the participants with concurrent hand grip exertion, at 5% or 40% of their maximum strength (low vs. high physical load), on a hand dynamometer. We found that visual search under physical effort is faster, but more vulnerable to distractor interference, potentially due to arousal and reduced inhibitory control, respectively. The two effects further manifest in different aspects of RT distributions that can be captured by different components of the ex-Gaussian model using hierarchical Bayesian method. Together, these results provide behavioral evidence and a novel model for two dissociable cognitive mechanisms underlying the effects of simple muscle exertion on the ongoing visual search process on a moment-by-moment basis.

Visual search, although a ubiquitous cognitive process in everyday life, is often studied in laboratory settings with precise control and manipulations of statically presented stimuli. However, real-world visual searches are often conducted in dynamic environments wherein observers actively interact with their surroundings. Such interactions can involve active information seeking with oculomotor or motor actions (e.g., manual reaching) that can subsequently affect visual attention (i.e., the “active vision” approach, Awh et al., 2006; Rizzolatti et al., 1994; Song & Nakayama, 2009). In addition, physical action often occurs concurrently and *parallelly* with cognitive processes. For instance, one may be looking for a gray compact car in a parking lot while carrying a heavy laptop bag or looking for a snack in a grocery store while carrying a heavy shopping basket. However, there is little research to address how visual attention operates in the real world and applied context like this. Given that action and cognition are often intertwined in everyday life (Mehta, 2016; Rosenbaum, 2017), it is thus pivotal to understand how *concurrent* physical action affects the ongoing cognitive processes, such as attention. Note, this is orthogonal to research regarding the *subsequent* effects of acute and chronic physical activities such as exercise on mood and cognition, which could be mediated by various exercise-induced psychophysiological changes (Knaepen et al., 2010; Tomporowski, 2003; Voss et al., 2013).

Some recent evidence suggests that concurrent physical activities can affect cognitive task performance; however, there is little consensus whether these effects are beneficial or detrimental (Huxhold et al., 2006; McMorris et al., 2009). Some studies showed negative effects of concurrent physical load, manifested as slower reaction time (RT) or reduced accuracy (i.e. *cognitive cost*, Hope et al., 2012), whereas others showed positive effects (i.e. *cognitive benefit*; Chang et al., 2012; Schmidt-Kassow et al., 2013) or mixed effects depending on the nature of the cognitive tasks (McMorris et al., 2011). These mixed findings make it difficult to conceptualize the impact of physical effort on cognition in a broad range of applied and translational settings.

To address some of these previous conflicting findings, we hypothesize that dissociable mechanisms, with potentially opposite behavioral effects, may jointly contribute to the effects of physical effort on visual attention. Effortful physical action with increased muscular activity and cardiovascular responses can trigger physiological arousal (Nielsen & Mather, 2015). It is well established that arousal and stress influence performance in a variety of cognitive tasks (Davey, 1973; Lee et al., 2018; Mather et al., 2016), following an inverted-U shaped relationship. That is, moderate levels of arousal facilitate performance as compared to low or high levels of arousal (Kleberg et al., 2019; Mather et al., 2020; Yerkes & Dodson, 1908). When applied to visual search, this *arousal hypothesis* would predict a search benefit with faster RTs under moderately high physical effort (Davranche et al., 2006; Droit-Volet & Berthon, 2017). On the other hand, physical effort may reduce inhibitory control, potentially due to competition for shared processes, such as the central executive process (Huxhold et al., 2006; Kurzban et al., 2013; Labelle et al., 2013; Woollacott & Shumway-Cook, 2002) between the concurrent physical and cognitive tasks. This *inhibitory control hypothesis* would predict detrimental effects of physical effort on visual search performance, for example, increased distractor interference when a successful suppression of task-irrelevant distractors is essential for task performance.

Our hypotheses are in general consistent with the large literature on the contributions of motor action on core cognitive processes, including the action-centered model of attention which underlines the role of action intentions in determining the way the environment is perceived (Lyons et al., 1999; Tipper et al., 1992) and how subsequent motor actions are planned accordingly (Cohen & Rosenbaum, 2004). For instance, Bekkering and Neggers (2002) showed that specific object features such as orientation and colors are differently prioritized depending on the type of actions, grasping and pointing, associated with the visual information. Similar findings were reported for different kinds of action intentions (Welsh & Pratt, 2008), such as power and precision grasp postures associated with visual sensitivity for temporal and detailed information, respectively (Thomas, 2015). This action-related feature weighting effect has also been extended in working memory such that size and color are better retained for grasping and pointing actions, respectively (Heuer & Schubö, 2017). The present study extends this literature in a novel direction toward the effects of physical actions on attention.

To test our working hypotheses, we conducted a dual-task experiment with a handgrip task and a singleton visual search task (Theeuwes, 1992) performed simultaneously on each trial (Fig. 1). In the handgrip task, participants squeezed a hand dynamometer with either low or high isometric muscle contraction until they made a speeded response for the concurrent visual search task. In the visual search task, participants made speeded button responses to report whether they found a pre-defined target in an array of visual stimuli. The target was a shape singleton (i.e., a unique shape) among distractors with a homogeneous but different shape. Critically, a salient-but-irrelevant distractor with a unique color (i.e., color singleton) was present on half of the trials but absent on the other half. Although this color singleton distractor is completely task-irrelevant, it can often capture spatial attention and subsequently interfere with the search process for the target (Theeuwes, 1992).

**Fig. 1:**
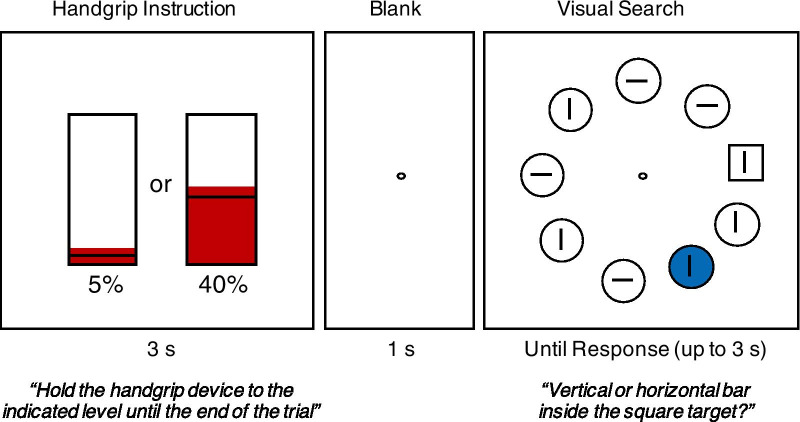
Illustration of the stimuli and task procedure for the handgrip–search dual task. Each trial began with the handgrip initiation to the required %MVC for 3 s with immediate visual feedback of exerted grip force shown on the screen. Participants were instructed to hold the grip force throughout the visual search task. For the visual search task, a search array appeared following a 1-s of center fixation and it stayed until the response (up to 3 s). The search array consisted of one target square and eight distractor circles evenly distributed on an imaginary circle. Each search item had a bar inside the outline shape. Participants were required to make a speeded response to report the orientation (vertical or horizontal) of the bar inside the target shape. On half of the trials, one of the circle distractors had a randomly chosen color background. This color singleton distractor often captures attention

There has been a heated debate whether salient distractor can involuntarily capture attention though never relevant (*stimulus-driven*; Franconeri & Simons, 2003; Theeuwes, 2010; Yantis & Jonides, 1984) or only information that matches predefined target feature or attentional set can capture (*goal-driven*; Folk et al., 1992). A recent signal suppression hypothesis as a hybrid model emphasizes the role of inhibition in avoidance of capture by salient distractors (for a recent review, see Gaspelin & Luck, 2018, 2019). According to this, a salient distractor can attract spatial attention, but it can also be actively suppressed by top-down control mechanisms that reallocate spatial attention to the designated target. The prominent role of the inhibitory control mechanism in visual search has been further supported by eye movement and event-related potential (ERP) studies using the distractor positivity (*P*_D_) ERP component as an index of active suppression (Gaspar & McDonald, 2014; Hickey et al., 2009; Sawaki & Luck, 2010, 2011). Given that the extent of inhibition against distractor interference can be dependent on the moment-by-moment availability of executive control process (Geng, 2014; Jiang & Chun, 2001; Mazaheri et al., 2011), the singleton distractor effect can thus be used as a behavioral measure of inhibitory control function available under concurrent physical effort. That is, the larger the singleton distractor effect under increased physical load, the lesser inhibition of the salient-but-irrelevant singleton distractor, as predicted by the inhibitory control hypothesis.

Importantly, the two mechanisms and their effects, the RT benefit by the arousal hypothesis and the RT cost due to increased singleton distractor effect by the inhibitory control hypothesis*,* are not necessarily mutually exclusive or independent of each other. The bidirectional RT effects are simply attributed to different cognitive processes that underlie the task performance. They may both rely on the same underlying neurocognitive mechanism at play, for example, effort-mediated phasic arousal (Aston-Jones & Cohen, 2005; Wetzel et al., 2012; Yerkes & Dodson, 1908). For the overall performance, however, they could jointly affect the overall RT and may cancel each other out, leading to a null result for averaged RT. On the other hand, the two effects may manifest in different aspects of RT. The expected faster RT from the arousal hypothesis could manifest as a leftward shift of RT distribution under moderately high physical effort (Davranche et al., 2006; Droit-Volet & Berthon, 2017). In contrast, the inhibitory control hypothesis predicts that the cognitive effects of concurrent physical effort on visual search would mainly manifest to the slowest portion of the RT distribution (i.e., right tail of the positively skewed distribution), given experimental manipulations or individual differences of executive function often affect slower RTs (McVay & Kane, 2012; Unsworth et al., 2010; West, 2001).

To precisely capture these potentially dissociable RT effects, we modeled RT distributions using an ex-Gaussian function with a hierarchical Bayesian method. The ex-Gaussian function is a convolution of a Gaussian distribution and an exponential distribution (see “Data analysis” section for detail). The ex-Gaussian model has been widely used in cognitive research to capture distinctive properties of experiment effects on RT distributions (e.g., Hohle, 1965). In contrast to point estimates of RT (e.g., mean or median RT) that inevitably discard some information, RT distributional analyses can be used to recover meaningful underlying sub-processes, potentially manifested differently on the shape of the RT distribution, especially in the context of visual search (Palmer et al., 2011; Wolfe et al., 2010). Note, our analytic approach here does not depend on assumptions or hypotheses regarding how the parameters of the ex-Gaussian model can be meaningfully mapped onto psychological processes (Park et al., 2017). Instead, the ex-Gaussian model was used in the present study as a descriptive or measurement model to (1) decompose the RT distributions into a Gaussian component and an exponential component; and (2) capture the hypothesized search benefit and cost of the concurrent physical load that are expected to manifest to dissociable distributional properties (i.e. benefit at overall RTs vs. cost at the long RTs). Specifically, the overall RT benefit for the arousal hypothesis should mainly manifest to both the Gaussian component of the ex-Gaussian function, whereas the cost in long RTs for the inhibitory control hypothesis should manifest to the exponential component.

In summary, the present study aimed to assess two dissociable mechanisms (arousal vs. inhibitory control) underlying the effects of effortful physical action on visual search in the face of a salient-but-irrelevant distractor singleton. Specifically, it was hypothesized that concurrent physical effort would facilitate visual search due to arousal, but slow down visual search due to reduced inhibition of the singleton distractor. The two effects would manifest to different aspects of RT distributions that can be captured by different components of ex-Gaussian modeling of the RT data.

## Methods

### Participants

Thirty volunteers (12 males) between 18 and 22 years old participated in each experiment for course credit at the University of California, Riverside. All had normal or corrected-to-normal visual acuity and reported having a normal color vision. Informed consent was obtained at the beginning of the experiment.

### Stimuli and procedure

Stimuli were presented on an LCD monitor with a homogeneous gray background (6.7 cd/m^2^) with a refresh rate of 60 Hz at a viewing distance of 57 cm, controlled with PsychToolbox (Brainard, 1997) in MATLAB (The MathWorks, Cambridge, MA) on a macOS operating system. The monitor was calibrated with an X-Rite I1Pro spectrophotometer (X-Rite, Inc., Grand Rapids, MI). A schematic illustration of the stimuli and task procedure is shown in Fig. 1. The experiment consisted of two-parts in sequence. The first part was a handgrip-only task measuring individual participant’s maximum muscular strength (maximum voluntary contraction; MVC) with subsequent handgrip practice trials for the participants to get familiarized with the hand dynamometer and handgrip exertions at different %MVC. The second part was the dual-task paradigm with the simultaneous handgrip task and singleton visual search task.

#### Part 1: Individual MVC and handgrip maintenance practice

MVC was measured for individual subjects at the beginning of the experiment. Participants were instructed to squeeze a hand dynamometer (model: HD-BTA; Vernier Software & Technology, Beaverton, OR, USA) using the left hand as hard as they could for 4 s. The exerted grip force was recorded in the unit of Newton with a 200-Hz sampling rate. This procedure was repeated three times with a few seconds of break between attempts. Individual MVC was then calculated as the mean of the median values of exerted force from each attempt.

Ten-trial handgrip practice then followed. Each trial started with a prompt “*Ready*” at the center of the display for 500 ms, followed by a visual gauge (for an example, see Fig. 1) indicating the required grip force (randomly chosen from 10%, 30%, 50%, 70%, or 90% MVC; each level repeated twice in a randomized order) at the current trial. The required grip force was indicated by the height of a black horizontal line in a black rectangular box (4° × 6° in visual angle). The height of the black horizontal line (proportional to the height of the box) was set at the required %MVC. Participants were instructed to hold and maintain the required force level over 4 s. The exerted grip force was indicated, in real time, as a red-colored bar on the visual gauge. If participants successfully maintained the required MVC for two-thirds the last 2 s out of the 4-s window (with 0–30% above the required %MVC in each condition; see below Part 2 for more detail), a display containing “*You have successfully maintained the force!”* with a “Cha-Ching!” sound would be presented for 1 s. Otherwise, a display of “*Not quite. Please try harder!*” with a “Beep” sound would be presented. Participants would receive another set of 10 trials if they failed for more than 50% of the practice trials.

#### Part 2: Handgrip–search dual task

The handgrip–search dual-task procedure is illustrated in Fig. 1. Each trial of the handgrip and search dual-task paradigm began with the identical handgrip task from the practice with two exceptions. First, the required handgrip muscle contraction was at either 5% (low physical load condition) or 40% (high physical load condition) of individual MVC, randomized across trials. This procedure made it possible to operationalize physical effort independent of individual differences in muscular strength and fitness level. Note, the low (5% MVC) and medium level (40% MVC) of physical exertion were selected for low and medium physical effort, respectively (Stevens & Cain, 1970). Second, the grip was maintained for three seconds instead of four seconds. Critically, the handgrip task was extended over the subsequent visual search task until a response was made. Note, no visual gauge of the exerted force was provided during the visual search task.

The visual search task started with a 1000-ms fixation circle on the blank screen, followed by the search array of nine white outline shapes (one target square and eight distractor circles; 2.2° × 2.2° in size with 0.08° line width) evenly distributed (40° apart) on an invisible circle with a radius of 6.2° from the center of the screen. Each outline shape contained a line inside (0.53° × 0.08°) in a vertical or horizontal direction selected randomly for each shape. Participants were instructed to search for the target item (the square shape) and make a speeded response to report the orientation of the line inside the target shape. The response was made using the two right shoulder buttons on a gamepad for vertical and horizontal target orientation, respectively, with the right hand while squeezing the dynamometer using the left hand.

On half of the trials, one of the circle distractors had a chromatic color background behind the white outline shape and inside line (i.e., singleton distractor present condition). The locations of the target and singleton distractor were randomly chosen, leading to random target–distractor spatial distance across trials. The color for the singleton distractor colors was also randomly selected on each trial from 180 colors evenly distributed on a continuous 360° circular color space (Zhang & Luck, 2008). All colors were in equal luminance but varied in hue and slightly in saturation. The randomization of the color for the singleton distractor was to prevent a potential reduction in distractor interference due to expectancy or adaption of a specific feature (Feldmann-Wüstefeld & Schubö, 2016; Theeuwes et al., 2000; Vatterott & Vecera, 2012; Won & Geng, 2018) if a fixed color is used for the singleton distractor.

The search array lasted up to 3000 ms and speeded response was heavily stressed but to the best of accuracy. Participants performed a total of 240 trials with 60 trials for each of the 2-by-2 conditions (i.e., high vs. low physical load and singleton distractor present vs. absent) divided into three blocks with self-paced long breaks in between. An additional set of short breaks was forced for every 40 trials to prevent physical fatigue.

One-second visual and auditory feedback was provided immediately after the search response. A visual message of “*Now Release… Both tasks are correct!”* and a “Cha-Ching!” sound would be presented if the responses for both tasks were correct (i.e. successful handgrip exertion until a correct search response to the search target was made). If either response or both responses were incorrect, feedback would then indicate the failed task (e.g. “*Handgrip/Search/Both failed!*”) with a “Beep” sound. To be classified as correct grip exertion, the exerted grip force on a given trial needs to be between 0 and 30% above from the required %MVC (i.e., 5–35% for the 5%MVC condition and 40–70% for the 40%MVC condition) for more than 66% of the measurement window that was defined as the last three-fourths of the time from the beginning of the handgrip task to the search response. This response classification criterion was quite conservative to avoid any unnecessary demands on fine motor control which would require heightened executive control (Guillery et al., 2017). Beyond this purpose, this criterion for the dichotomous classification of handgrip accuracy was largely arbitrary. We thus also calculated a continuous measure of exerted grip force at the trial level as the median of continuously recorded force exertion during the visual search on each trial. This measure would allow us to assess how individual differences in actually exerted handgrip force affect the search performance.

### Data analysis

For raw RT analyses, only the correct RTs for both the visual search task (accuracy: 96.5%, [95% CI 95.1%, 97.8%]) and the handgrip task (accuracy: 97.6% [96.6%, 98.6%]) were included (Table 1). This resulted in a loss of 5.6% of trials averaged across all participants. No trial-level RT outlier rejection was performed given that the primary data analysis was modeling RT distributions using a hierarchical Bayesian method which is robust to outliers (Rouder et al., 2003, 2005).

**Table 1:** Mean accuracies and correct reaction times (RTs) from each experimental condition

Singleton distractor	Accuracies (%)		RTs (ms)	
	Low load (5%)	High load (40%)	Low load (5%)	High load (40%)
Absent	98.4 ± 0.3	98.6 ± 0.3	730.0 ± 30.9	688.6 ± 23.2
Present	97.5 ± 0.9	97.2 ± 0.9	779.8 ± 31.6	758.4 ± 25.5

For RT distributional analyses, we used the ex-Gaussian function to model correct RTs from each condition and each participant, with the hierarchical Bayesian approach. The ex-Gaussian model assumes that RT distributions are drawn from a mixture of Gaussian and exponential distributions, and has been widely used to model RT datasets from various cognitive tasks (Balota & Yap, 2011; Heathcote et al., 1991; Ratcliff & Murdock, 1976). While one-on-one mapping between ex-Gaussian model parameters and specific cognitive processes is so far controversial (Matzke & Wagenmakers, 2009), there are at least some consensus (Balota & Spieler, 1999; Schmiedek et al., 2007; Spieler et al., 1996). Specifically, the Gaussian component with two parameters mu (*μ*) and sigma (*σ*) is considered to reflect, but not exclusively, stimulus- or response-related processes as its effect on the shape of the RT distribution is analogous to a non-decision time (*T*_er_) in drift–diffusion models (Schmitz & Wilhelm, 2016). The exponential component with a single parameter tau (*τ*) is often sensitive to central attentional processes, especially those that require inhibitory control (McAuley et al., 2006; Shao et al. 2012; Spieler et al., 1996).

The ex-Gaussian parameters were estimated using the hierarchical Bayesian method (Rouder et al., 2005) with MatlabStan (Stan Development Team, 2016). This approach has several advantages over conventional single-level maximum likelihood estimation procedure (for details see Rouder et al., 2014). For instance, the hierarchical Bayesian model can estimate a range of population-level parameter posteriors with bidirectional shrinkage between upper and lower-level parameters. As a result, it can account for multiple sources of variability simultaneously. With this approach, we estimated the main effects for each population-level parameter by sampling from the Normal distribution where the mean is a sum of the fixed effect (condition-level) and the random effect (individual-level), and the interaction effect describes the individual-by-condition variability, as in a general linear model (Rouder et al., 2014). We chose reasonable to non-informative priors for all parameters that cover possible values within a plausible range of our RT dataset (e.g., 0–5 s) to prevent bias of the posterior distribution by the choice of the priors.

A total of 40,000 samples were drawn after 40,000 warming-up samples from four Markov Chain Monte Carlo (MCMC) chains. Model convergence was assessed by an *R̂* value close or equal to 1.00 for all parameters (Gelman & Rubin, 1992). Statistical inference was made based on comparisons of the mean and 95% highest density interval (HDI; Kruschke, 2011) of the posterior distributions of the group-level parameters between experimental conditions. The HDI represents the smallest interval of parameter values covered by 95% of the posterior density. That is, we can be 95% certain in probability that the true parameter value lies within the HDI. The HDI can thus be considered as a Bayesian alternative to confidence intervals but with different statistical assumptions. Because the uncertainty or the strength of evidence is reflected in the spread of the posterior distribution, when applied in a comparison between conditions, we can credibly reject the null hypothesis if the HDIs from the parameter posterior difference between conditions do not include zero (Kruschke, 2014). For the consistency, we provided Bayes factors (BFs) for other statistical results under the frequentist null hypothesis significance testing (NHST). For example, BF_10_ quantified the evidence in favor of a given alternative hypothesis (*H*_1_) against the prior null hypothesis (*H*_0_), and the value of BF_10_ indicates how many times the alternative hypothesis is more likely than the null (e.g., BFs greater than 3 are generally regarded as providing substantial evidence of one model over the other).

## Results

### Correct reaction time

**Fig. 2:**
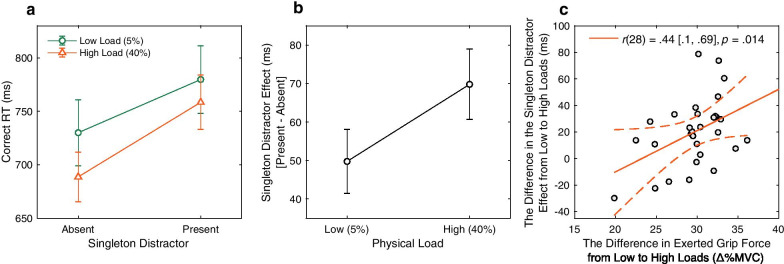
RT results. **a** Mean RTs from trials with correct responses for both tasks across the singleton distractor present and singleton distractor absent conditions, plotted separately for low physical versus high physical load conditions. **b** Singleton distractor effects were assessed as the difference in RTs between singleton distractor present and absent conditions. The high physical load condition resulted in a larger distractor interference effect. **c** The difference in the singleton distractor effect from low to high loads (i.e., the interaction effect in **a**; see main text for detail) significantly correlated with the difference in exerted grip force from low to high loads (Δ_%MVC_: high–low load) across the participants. The solid and dashed lines represent the best linear fit and its 95% confidence interval, respectively. Pearson correlation coefficient (*r*) with 95% confidence interval and *p* value are presented in the figure. Error bars in **a** and **b** represent standard error of mean

As shown in Fig. 2a, the mean correct RT seems larger at the singleton distractor present condition than the singleton distractor absent condition, and larger under low physical load than high physical load. Consistent with these observations, a repeated-measures two-way analysis of variance (ANOVA) with two factors of the singleton distractor presence (absent vs. present) and the physical load (low load vs. high load) revealed significant main effects of the singleton distractor presence, *F*(1, 29) = 50.51, *p* < 0.001, *η*_*p*_^2^ = 0.635, BF_inclusion_ > 1000, as well as the physical load, *F*(1, 29) = 8.07, *p* = 0.008, *η*_*p*_^2^ = 0.218, BF_inclusion_ = 93.96. The main effects suggested that visual search slowed down by the presence of the irrelevant color singleton, potentially due to attentional capture by the singleton distractor (Theeuwes, 1992), but speeded up under high physical effort, as predicted by the arousal hypothesis. Importantly, a significant interaction was found, *F*(1, 29) = 18.35, *p* < 0.001, *η*_*p*_^2^ = 0.388, BF_inclusion_ = 1.90, which was largely driven by the increased singleton distractor cost (present–absent; see Fig. 2b) under the high load condition (69.8 ms [51.8 ms, 87.7 ms]) compared to that under the low load condition (49.8 ms [33.5 ms, 66.0 ms]), *t*(29) = 4.28, *p* < 0.001, Cohen’s *d* = 0.79, BF_10_ = 149.56. These results indicate that, as predicted by the inhibitory control hypothesis, the concurrent physical effort increased interference, due to more attention capture by the irrelevant color singleton distractor.

The degree to which physical effort that the handgrip maintenance task would create varied with required physical load, identified by handgrip accuracy difference between low load (99.6% [99.2%, 100.0%]) and high load (93.3% [90.7%, 95.9%]), *t*(29) = 4.76, *p* < 0.001, Cohen’s *d* = 0.88, BF_10_ = 491.41. Therefore, this gives rise to a possibility that the results from search RTs may partially reflect stronger subjects’ motivation to terminate the search under the high load condition (i.e., faster response under 40% MVC physical load) given that each trial in the handgrip–search dual task ended as soon as the visual search response was made. This strategy would likely lead to speed-accuracy trade-off in search performance. To assess this possibility, we performed a repeated-measures two-way ANOVA for accuracy (see Table 1). This analysis revealed that neither the main effects of the singleton distractor presence, *F*(1, 29) = 1.64, *p* = 0.211, *η*_*p*_^2^ = 0.053, BF_inclusion_ = 0.73, and the physical load, *F*(1, 29) = 1.64, *p* = 0.211, *η*_*p*_^2^ = 0.053, BF_inclusion_ = 0.15, nor the interaction effect between them was significant, *F*(1, 29) = 0.74, *p* = 0.389, *η*_*p*_^2^ = 0.026, BF_inclusion_ = 0.09. These results thus provide some preliminary evidence against the alterative motivation-based account.

Note, the exerted grip force showed large variance across participants (low physical load condition: 12.0% MVC [11.1%, 12.9%], with the range from 8.2 to 20.2% MVC; high physical load condition: 41.7% MVC [40.4%, 43.0%], ranged from 30.8 to 50.0% MVC), with significant overshooting at the low load condition (i.e., 5% MVC), *t*(29) = 15.03, *p* < 0.001, Cohen’s *d* = 2.79, BF_10_ > 1000. With these large individual differences in exerted grip force across participants, the exerted force was significantly higher at high physical load as compared to low physical load, *t*(29) = 44.70, *p* < 0.001, Cohen’s *d* = 8.30, BF_10_ > 1000, confirming the validity of the physical load manipulation. Moreover, the measure for the exerted grip force provides a novel way to assess the relationship between grip exertion and visual search performance without the dichotomous classification of handgrip accuracy. Specifically, we looked at the relationship between the differences in the exerted grip force between the two load conditions (Δ_%MVC_: 29.7%, [28.4%, 31.0%], ranged from 19.9 to 36.1%) and the differences in the color singleton effect (Fig. 2b) between the two load conditions across all participants. As shown in Fig. 2c, participants who showed larger differences in exerted grip force between the load conditions tend to have larger differences in the color singleton effect between the two load conditions. That is, individual Δ_%MVC_ was significantly correlated with the interaction effect of the two factors (i.e., the physical load effect and the singleton distractor effect)*, r*(28) = 0.44 [0.10, 0.69], *p* = 0.014, BF_10_ = 3.95, suggesting that distractor interference increased as the effective physical load increased across participants.

### Hierarchical Bayesian model of RT distributions

In summary of the point estimate RT analyses, we found that visual search became slower not surprisingly when the singleton distractor was present, whereas it became faster when the physical load was higher. Moreover, the interaction of the two factors revealed an increased distractor cost under higher physical load, which was further positively correlated with individual Δ_%MVC_ across participants. To further identify the characteristics of the RT benefit and cost effect of the concurrent physical load, and specifically to see if they would potentially result from different mechanisms, distributional analyses were performed on the RT data.

**Fig. 3:**
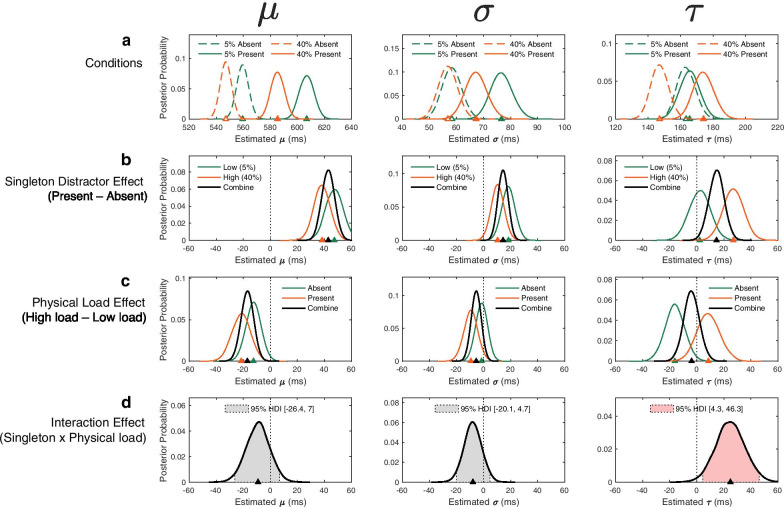
Posterior distributions of the hierarchical Bayesian ex-Gaussian parameters. The curves indicate the fits of a nonparametric kernel density estimates to the posterior samples for each group-level parameter. The triangles on the x-axis indicate posterior means. The posterior distributions of each parameter across all four conditions (**a**) can be decomposed into the main effect of singleton distractor presence versus absence (**b**), physical load (**d**), and the interaction effect (**d**). For the interaction effect (**d**), shaded areas represent 95% highest density interval (95% HDI) of posteriors. Only the exponential parameter *τ* yielded a credible interaction effect with the lower bound not crossing zero

We fit the ex-Gaussian function to the correct RTs using hierarchical Bayesian method. With this method, we estimated group-level posterior distributions of parameters for ex-Gaussian function (see “Data analysis” for detail), including *μ* and *σ* for the Gaussian component and *τ* for the exponential component (Fig. 3a). From the overall posterior distributions of the ex-Gaussian population-level parameters, we reconstructed the main effects of singleton distractor presence versus absence (Fig. 3b) and the physical load (Fig. 3c) and the interaction effect (Fig. 3d).

First of all, the most distinctive pattern can be found between the distractor absent and present conditions in all three parameters (Fig. 3b). Mean posterior with 95% HDI revealed that the singleton distractor effect was credibly in the positive direction, indicating slower speed, in all three ex-Gaussian parameters: *μ* (+ 42.8 ms, 95% HDI [+ 34.2 ms, + 51.1 ms]^1^), *σ* (+ 14.4 [+ 8.0, + 20.3]), and *τ* (+ 14.6 [+ 4.1, + 25.0]). This 95% HDI indicates that there is a 95% certainty that a credible range of RT difference captured by each parameter lies within their lower and upper bound. Therefore, the positive posterior means and their positive HDIs (not crossing zero to negative) indicated credible delays in both Gaussian and exponential components of the RT distribution due to distractor singleton, replicating the classic attentional capture effect. These results are similar to some previous findings on the Stroop compatibility effect using the ex-Gaussian function (Mewhort et al., 1992; Spieler et al., 1996).

The physical load effect (Fig. 3c), on the other hand, was largely captured by a decrease in *μ* (− 17.0 [− 25.3, − 8.6]), but not in *σ* (− 5.4 [− 11.4, + 0.8]) or *τ* (− 3.8 [− 14.7, + 7.0]). It suggests that the mean RT benefit from the high physical load condition was driven by overall faster responses across all proportions in the RT distribution. This finding is consistent with our prediction that the arousal-driven RT benefit would manifest as a general leftward shift of the RT distribution, similar to previous findings (e.g., Davranche et al., 2006).

**Fig. 4:**
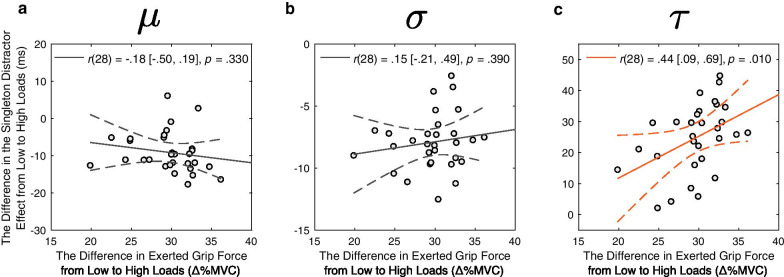
The interaction effects and the difference in exerted grip force. The difference in the singleton distractor effect from low to high loads (i.e., the interaction effect in Fig. 3d) in *τ* (**c**), but not in *μ* (**a**) or *σ* (**b**), significantly correlated with the difference in exerted grip force from low to high loads (Δ_%MVC_: high–low load) across the participants. The solid and dashed lines represent the best linear fit and its 95% confidence interval. Pearson correlation coefficient (*r*) with 95% confidence interval and *p* value are presented in the figure

Importantly, the interaction effect observed in the raw RT data analyses mainly manifested to the exponential component (*τ*: + 24.8 [+ 4.3, + 46.3]), but not the Gaussian component parameters (*μ*: − 9.1 [− 26.4, + 7.0]; *σ*: − 7.9 [− 20.1, + 4.7]), as shown in Fig. 3d. This finding was further supported by a significant correlation between the differences in the exerted grip force between the two load conditions (Δ_%MVC_) and the differences in the color singleton effect on *τ* between the two load conditions, *r*(28) = 0.44 [0.09, 0.69], *p* = 0.010, BF_10_ = 3.91. In contrast, neither of the Gaussian parameters showed this relationship [*μ*: *r*(28) =  − 0.18 [− 0.50, 0.19], *p* = 0.330, BF_10_ = 0.35; *σ*: *r*(28) = 0.15 [− 0.21, 0.49], *p* = 0.390, BF_10_ = 0.32]. It is thus possible that the correlation results in the raw RT analyses between the individual Δ_%MVC_ and the interaction effect (Fig. 2c) were primarily driven by the *τ* effect (Fig. 4c). This possibility was confirmed by a mediational path analysis. Specifically, the significant association between Δ_%MVC_ and the interaction effect in raw RT (total effect: *β* = 0.13 [0.02, 0.22], *p* = 0.008) was fully mediated by the exponential component *τ* (the direct effect: *β* = 0.02 [− 0.05, 0.09], *p* = 0.434, versus the indirect effect: *β* = 0.11 [0.04, 0.21], *p* = 0.014).

## Discussion

Given the prevalent action-cognition interaction in everyday life, identifying how simple physical actions affect ongoing cognitive processes could be a big step toward understanding cognitive functioning in everyday life. The present study aimed to assess how physical effort (handgrip exertion) affects visual attention and inhibitory control in a dual-task paradigm with concurrent visual search and handgrip tasks. We found that visual search became faster in general under higher physical load (grip force exertion at 40% MVC) than lower physical load (at 5% MVC), potentially due to the medium level of arousal from hand grip. In contrast, higher physical load increased the interference from the singleton distractor and resulted in less efficient search, reflecting reduced inhibitory control under high physical effort. This increase in interference from the singleton distractor from low to high physical load significantly correlated with the increases in exerted grip force from low to high physical load across participants (Δ_%MVC_).

These findings were further supported by ex-Gaussian fits of RT distribution data using hierarchical Bayesian method. Specifically, the beneficial effect of physical effort on visual search was exclusively driven by the Gaussian mean component (*μ*), whereas the detrimental interference effect (i.e., the interaction effect) largely manifested to the exponential component (*τ*). Furthermore, the individual differences in the increased interference from low to high physical load could be largely accounted for by the increases in τ for the exponential component of the RT distribution, but not by the Gaussian component (*μ* and *σ*). These findings are consistent with the proposed dissociable mechanisms that can cancel each other out in overall RTs but manifest in distinct distributional properties of RTs identified by the hierarchical Bayesian posterior ex-Gaussian parameter distributions. These findings add to the growing literature demonstrating how effective RT distributional analyses can be used to disentangle underlying cognitive processes (Spieler et al., 2000).

Together the results provide strong support for the dissociable effects of physical load on visual search. That is, visual search under physical effort is generally faster, but at the same time vulnerable to distractor interference. The present study thus provides a potential account of mixed findings in the literature regarding the effect of concurrent physical effort on cognition.

This research could facilitate our understanding of cognition-action interaction in the domain of visual attention, a potentially significant contribution to the emerging research on filling the large gap in our knowledge about how visual processing actively interacts with the observers and the external world in everyday life (see for review, Brady et al., 2019). There has been some work on *stimulus*-oriented factors to account for modulations of attentional allocation by semantic and episodic knowledge (Chen & Zelinsky, 2006; Park et al., 2015; Störmer et al., 2019; Yang & Zelinsky, 2009), reward-association (Anderson et al., 2011), and contextual information (Castelhano & Henderson, 2007; Oliva & Torralba, 2001; Wolfe et al., 2011). Other studies have investigated the impact of *state*-oriented factors (e.g. observer’s mental and physical states at the moment of the search). For instance, the influence of the affective state on attentional processes has largely been studied (Jefferies et al., 2008; Olivers & Nieuwenhuis, 2005). Nonetheless, there is little research on the effects of moment-by-moment physical actions on ongoing search processes.

The dissociable RT distribution effects observed in the present study are consistent with some previous findings. The overall leftward shift of the RT distribution due to physical effort (and likely induced arousal) in the present study is in line with some previous research showing leftward RT shift as a consequence of physical exercise in a choice RT task (Davranche et al., 2006) or emotional arousal in an implicit timing task (Droit-Volet & Berthon, 2017). On the contrary, the RT cost with the singleton distractor, potentially due to reduced inhibitory control under increased physical load, mainly manifested in the slowest RTs. This is consistent with the worst performance rule (Larson & Alderton, 1990). That is, the slowest RTs on a cognitive task are most predictive of higher-level cognitive function, especially executive control and fluid intelligence (Unsworth et al., 2010). Consequently, these RT effects are often associated with the ex-Gaussian *τ* parameter (Ratcliff et al., 2008; Schmiedek et al., 2007).

Our adoption of the hierarchical Bayesian approach may be critical to assess the cognitive effects of concurrent physical effort for two reasons. First, distributional analyses of RT data, with a relatively large degree of individual-level variability, often require a large number of observations per condition and participant. This requirement is difficult to meet since a large number of trials with physical exertions would lead to physical fatigue. However, the hierarchical Bayesian method can drastically reduce the number of trials, because estimates of subject-level are further informed by group-level parameters (Rouder & Lu, 2005). Second, Bayesian parameter estimation provides not only the best point estimate of a parameter but also a credible range of values and the precision of estimation (as reflected in the HDI of the posterior distributions). This feature is thus highly sensitive to small-to-medium effects such as some of the operations in the present study.

Future research needs to elucidate the neural mechanisms for the costs and benefits of physical exertion on ongoing cognition. Although the arousal and inhibitory control processes seem to drive the observed search benefit and cost independently, it does not necessarily mean that they are supported by fundamentally different neural mechanisms. The current findings may fit well with the dual-task cost framework (Kahneman, 1973). In light of recent evidence that cognition and action may recruit overlapping brain regions (and potentially common cognitive processes and resources, Ballard et al., 1997; Leisman et al., 2016), it is plausible that the conventional within-domain dual-task costs may extend to cross-domain dual-task paradigms such as the handgrip–search tasks in the present study.

The exact source of this cross-domain dual-task cost is not yet clear. It could be closely related to the cost and benefits of effort in general (Kurzban, 2016), due to the impact of effort on metabolic processes and executive control. Alternatively, it may be driven by the close relationship between physiological arousal and attentional system (Kahneman, 1973). The classical Humphreys and Revelle model (1984) of arousal and performance emphasizes the nature of cognitive task demand as a determining factor for whether arousal will have a positive or negative effect on different processes (Callejas et al., 2005; McConnell & Shore, 2011). It is plausible that both the benefit and cost effects could originate from increased arousal, but manifest in a hierarchical manner. That is, moderate physical arousal speeds up overall information processing, which, depending on task context, can be coupled with changes in distractor interference (Wetzel et al., 2012; Yerkes & Dodson, 1908). The latter is in principle consistent with the involvement of the locus coeruleus–noradrenaline (LC–NE) pathway in arousal mediated effects on distractor interference (Aston-Jones & Cohen, 2005). Relatedly, previous studies demonstrated that responses with shorter latency are more susceptible to interference from distractors than slower response-directed movement initiation (van Zoest & Donk, 2008; van Zoest et al., 2004).

This arousal-based account is further related to some other alternative mechanisms specific to the context of additional singleton search paradigm that could account for the increased distractor interference under the concurrent physical load in the current study. One possibility is that exertion of physical effort leads to arousal and more importantly higher vigilance and readiness against *any* salient information across the visual fields regardless of its task relevance (Aston-Jones & Cohen, 2005; Nieuwenhuis et al., 2005). In our task, this may engage participants into the singleton detection mode (Bacon & Egeth, 1994) in which they are willing to accept greater capture by singleton distractor in exchange for reduced cognitive effort (i.e., deliberate evaluation of targetness for the salience signal). Similarly, an increase in arousal due to physical exertion may affect participants’ motivation and thus encourage them to make strategic responses to terminate the search task sooner. This arousal-based motivation effect would be assumed to reflect a shift of internal decisional criterion (e.g., signal detection criterion or boundary separation of evidence accumulator), so that relatively less amount of sensory evidence would satisfy the decision threshold (Bowen et al., 2016; Ratcliff & Tuerlinckx, 2002). Consequently, it could lead to the same *faster-but-vulnerable* search we observed. Although we did not find the indication of speed-accuracy trade-off from our data, the ceiling level performance (overall accuracy at around 98%) might have diluted the likelihood of this motivation-related response strategy. A more direct way to examine this motivation effect could be done by equating a total duration of physical exertion across physical load condition, regardless of search response time (e.g., hold 3 s from the search array onset).

Another alternative explanation for the relatively reduced singleton distractor cost under lower physical load could be raised from the idea of dimensional weighting account for attentional capture by singleton distractor (Müller et al., 2009). According to this idea, up-regulation of a relevant feature or a particular target shape in one condition but not in the other condition could explain less interference from the irrelevant feature dimension (i.e., color singleton), rather than better inhibition to the distractor. However, this dimensional weighting account would not be compatible with other results of our study. Regardless which of low or high physical load conditions this up-regulation of target feature is adopted, it should predict faster RTs irrespective of distractor presence. As shown in Fig. 2a, RTs from the distractor-*absent* trials were generally faster under high load which does not fit well with the increased distractor interference, and vice versa for the low load condition which showed relatively smaller distractor interference but longer RTs in responding to a target shape. The primary results pattern is pointing to the bi-directional effect of physical load manipulation, that is, the way physical load influences visual search is rather opposite depending on the presence of singleton distractor.

A key difference of these alternative explanations with ours is the role of active inhibition in distractor rejection and its association to concurrent physical effort. Further research with eye-tracking or electroencephalogram measures could obtain several advantages to resolve whether increased distractor interference under higher physical effort is a result of reduced inhibitory control function (Gaspelin & Luck, 2018). Oculomotor suppression through a bias in the first saccade or trajectory (Gaspelin et al., 2017; see also for reaching trajectory deviation in attentional capture paradigm, Moher et al., 2015; Welsh & Elliott, 2004) or the ERP *P*_D_ component with a comparison to the N2pc component, an electrophysiological index of the covert deployment of visual attention (Burra & Kerzel, 2014; Gaspar & McDonald, 2014) could be useful candidate measures. A correlation of such indices of inhibitory function with arousal level indexed by the pupil size (Gilzenrat et al., 2010; Zénon et al., 2014) could provide compelling evidence attributing increased distractor interference induced by physical load to the failure of inhibitory control, rather than hyper-vigilance or motivation accounts.

It remains unanswered whether or how our findings can be generalized for other types of physical effort such as cardio exercises, including several previous studies having participants walking or running on a treadmill or cycling (see for a comprehensive review, Chang et al., 2012). Considering the influence of specific action intentions on visual attention processing (Bekkering & Neggers, 2002; Tipper et al., 1992), even a perceptually equivalent amount of physical effort might create different cognitive consequences depending on what one’s physical/motor state is meant to deal with the environment. That is, categories of motor tasks defined by some distinctive elements such as use of body parts (e.g., reaching vs. walking) or intentions (e.g., pointing vs. grasping) might constrain how the associated visual processing is modulated (Rosenbaum et al., 2013). In addition, our handgrip maintenance task demanded both exertion of physical force and general effort; thus, it is yet conclusive whether the observed distractor interference effect is purely due to physical exertion or a combination of exertion and effort. Further attempts to answer this would be essential in promoting generalizability to real-world implications and a broader theoretical understanding of how physical action interacts with attention.

Understanding how physical actions influence cognition and the underlying neurocognitive mechanisms has great potentials to be applied broadly in society. Underperforming core cognitive functions under concurrent physical efforts can influence many aspects of our lives. The present research can shed light on the factors that underlie the interactions between physical and mental activities and address the extent to which some cognitive processes may, and others may not, be immune to physical strain. This can lead to a better understanding of the interactions between physical and cognitive activities in a broad range of domains, including education, ergonomic and work productivity, human–computer interfaces, defense and military, and sports. For instance, adaptive increases in the grip force (GF) in microgravity (Opsomer et al., 2018), additional physical efforts during extravehicular activity (EVA), reduced muscle strength over spaceflight missions (Winnard et al., 2019) could impair core cognitive processes, which could reciprocally reduce astronauts’ sensorimotor functions (Bray et al., 2012). Together, these negative effects of physical effort pose a unique challenge and further necessitate development of strategies to account for suboptimal decision-making due to physical strain.

## Conclusions

The current study provides behavioral evidence for two dissociable cognitive processes underlying the effects of simple muscle exertion on the ongoing visual search process on a moment-by-moment basis. In summary, our results suggest that cognitive processing can be modulated when concurrently engaged in a physically effortful state, and such effect can manifest as a benefit in RT but depending on task demand it can also come with a cost of being prone to interference. It contributes to the research theme of this special issue by understating how attention and executive control operate under effortful physical actions, which can occur frequently in everyday life. When applied in real-world issues, our results would help to develop a performance and safety guideline in workplaces where people manage to deal with both physical and mental activity.

## Data Availability

The datasets generated for the current study are available upon request from the first author.

## Footnotes

1 Hereafter, the mean and the 95% HDI were in the unit of millisecond. The positive and negative signs represent its direction.
